# A Case Report of COVID-19 in a Living Kidney Transplant Recipient: A Challenging Case with Complete Rehabilitation

**DOI:** 10.1155/2022/2517674

**Published:** 2022-01-17

**Authors:** Mahtab Poor Zamany Nejat Kermaney, Mohammad Hamidi Madani, Milad Bonakdar Hashemi, Alireza Zadmehr, Mehdi Dadpour, Fatemeh Roodneshin

**Affiliations:** ^1^Department of Anesthesiology, Shahid Beheshti University of Medical Sciences, Tehran, Iran; ^2^Resident of Urology, Labbafinezhad Hospital, Urology Nephrology Research Center (UNRC), Shahid Beheshti University of Medical Sciences, Tehran, Iran; ^3^Urology and Nephrology Research Center, Shahid Labbafinezhad Hospital, Shahid Beheshti University of Medical Sciences, Tehran, Iran

## Abstract

Adjustment of immunosuppressive and COVID-19 treatment in terms of drug interactions is still challenging. Herein, we report a 45-year-old woman with end-stage renal disease due to autosomal dominant polycystic diseases (ADPKD) with COVID-19 and pulmonary involvement following kidney transplantation. The patient was properly treated by discontinuation of immunosuppressive drugs, bronchoscopy, and high volume of blood transfusions. The fact that we quickly used early intubation and a new treatment regimen that suppressed immune systems may help physicians develop optimal treatment strategies for similar severe cases. However, this treatment method requires more detailed evaluations due to the contradictory results in reviewing other studies.

## 1. Introduction

In late December 2019, China officially declared several cases of severe acute respiratory disease in humans, leading to a pandemic by novel coronavirus SARS-CoV-2.1 [1]. The epidemic coronavirus disease rapidly spread worldwide; on 11 March (2020), it was officially stated a pandemic by the World Health Organization (WHO) [2]. By May 1, 2020, around 247,000 people deaths resulting from COVID-19 were recorded worldwide [3]. It is assumed that RNA beta coronavirus enters the cell and impacted the function of angiotensin-converting enzyme 2 (ACE2) receptors present in the lungs, kidneys, heart, and vascular endothelium [4]. The clinical presentations of infected patients vary from mild symptoms, for instance, flu-like symptoms, including fever, cough, and also shortness of breath, to severe and advanced diseases; it was demonstrated that acute respiratory distress syndrome may result in cardiopulmonary collapse [5]. There are several underlying health conditions such as chronic respiratory diseases, chronic immunosuppression, diabetes mellitus, and hypertension that make patients vulnerable to infectious diseases [6]. Immunosuppressive therapy in kidney transplant recipients including antilymphocyte therapy, a calcineurin inhibitor (CNI, e.g., tacrolimus), high-dose corticosteroids, and mycophenolate mofetil (MMF) may have influence on health status making patients more susceptible to COVID-19 [7, 8]. Consequently, adjustment of immunosuppressive and COVID-19 treatment in terms of drug interactions is still challenging. Herein, we report a case of COVID-19 with pulmonary involvement following kidney transplantation, which was properly treated by discontinuation of immunosuppressive drugs, bronchoscopy, and high volume of blood transfusions.

## 2. Case Presentation

A 45-year-old married woman with end-stage renal disease due to autosomal dominant polycystic diseases (ADPKD) with a history of routine hemodialysis three times a week was a candidate for receiving a donated kidney from his spouse and underwent living-related kidney transplantation on February 18, 2020. The patient's clinical laboratory outcome before her kidney transplantation is revealed in Table 1. Her previous medical history (including hypertension, diabetes mellitus, coagulopathy, and elective surgery) was negative. The donor was the patient's husband. Both the recipient and the organ donor had no previous international travel or exposure to contaminated or suspected patients infected with COVID-19. A preoperative ultrasound and abdominopelvic CT scan of the patients' native kidneys revealed high-volume native kidneys and confirming ADPKD. As a result, it was decided that the patient should undergo simultaneous unilateral nephrectomy and living-related kidney transplantation.

Since then, on February 18, 2020, she successfully underwent living-related kidney transplantation and nephrectomy surgeries. Throughout postoperative monitoring in the recovery ward at the first evaluation, the patient developed an episode of hypotension and tachycardia. The patient was immediately taken back to the operating room for exploratory surgery with the surgery team's opinion. During exploratory surgery, it was revealed that there are multiple areas of bleeding despite adequate blood coagulation. These bleeding areas were treated with packing lap sponges, which remained in the field for two days. After that, the patient was taken to the intensive care unit (ICU), and she remained intubated due to hypoxia. For routine checkups after surgery, in the emergency tests, acidosis was detected (Table 1). The patient was started on an appropriate immunosuppressive regimen, consisting of tacrolimus (1 to 1.5 mg per 12 hours and the concentration remained 4.0 to 5.0 ng/mL), mycophenolate mofetil (250 mg every 12 hours), and prednisolone (50 mg daily). The initial ventilator settings were TV = 500 ml/kg/FIO_2_ = 90%/RR = 20-30, which were adjusted over the following days based on the patient's clinical condition and laboratory tests. Lap sponges were removed two days later, and she was still connected to a ventilator, and O_2_ saturations were around 92%. On July 22, a decrease in oxygen saturation was detected. Despite the tracheal tube replacement, the O_2_ saturation was still about 83%, blood pressure was 120/80 mmHg, and no other findings like fever were detected (Table 1).

As a consequence, the patient underwent diagnostic and therapeutic bronchoscopy after consulting with a pulmonologist. During the procedure, copious amounts of clotted blood and mucus were removed. A bronchoalveolar lavage (BAL) sample was submitted for further examination; real-time reverse transcriptase-polymerase chain reaction (RT-PCR) of the BAL sample was confirmed positive COVID-19. In addition, the other symptoms, such as fever (37.5 to 38.9°C), frequent cough, breathlessness, diarrhea, and chest computed tomography (CT) (multiple patchy ground-glass opacity and exudative lesion in bilateral lungs), were confirmed. The next day, a second identical bronchoscopy was performed, and the patient's oxygen saturation improved to 90%. Two days later, the patient felt better, with a reduced body temperature (37.1°-36.2°C) and no longer having breathlessness or cough.

Therefore, the patient was extubated. As a result, all immunosuppressive treatments were discontinued, and an infectious disease specialist consultation was conducted to decide the following line of treatment. At the time of the onset of the COVID-19 pandemic, there was no specific protocol for discontinuing immunosuppressive regimen in transplant patients when needed. In this case, we discontinued mycophenolate mofetil, immediately. Tacrolimus was reduced and stopped within 3 days. Prednisolone, which was started at a dose of 50 mg, was reduced to a dose of 5 mg.

The patient received 20 units of packed cell and 25 fresh frozen plasma units (FFP) and 15 units of platelets for four days following surgery.

The patient was dismissed from the hospital on 30 July 2020. From that day forward, she was undergoing regular blood tests. This patient had a routine examination three weeks after being discharged from the hospital, and no fever or other symptoms were reported. The patient lost 8 kg of body weight during hospitalization. It is interesting to note that despite temporary discontinuous of immunosuppressive drugs, the function of the graft kidney is good after one year of follow-up. Serum creatinine is 1.1 mg/dl, and GFR is estimated 63 ml/min/1.73 m^2^. The patient's written consent was obtained to report the case descriptions.

## 3. Discussions

A novel coronavirus (COVID-19) that accompanied significant morbidity and mortality, as newly recognized in late December 2019 in Wuhan, China, is spreading worldwide [9, 10]. In this study, CT chest [11], fever, cough, RT-PCR positive of a sample of the lavage, shortness of breath, and diarrhea are close to the symptoms in the general population of severe COVID-19 pneumonia [12, 13]. Diagnosis mostly depends on radiological findings, laboratory results, and history taking. Initially, symptoms can be subtle, particularly in the early phases consisting of fever and cough with unremarkable lab test results [14]. It is also said to ground pneumonic glass opacities in chest tomography which is highly suggestive [5]. Despite up to 30% of false-negative results stated for the PCR tests from oropharyngeal/nasal swab samples, it is still one of the best methods for detecting suspicious cases [5]. Moreover, we can perform repeated oropharyngeal/nasal swabs or taking bronchoalveolar lavage if the tests are negative. However, clinical indicators like chest tomography scans can illustrate an urgent need for isolation in suspected cases [15].

Recent research suggests that an early intubation strategy is critical for covid19 patients [16, 17]. Thus, the findings of this study are consistent with previous research and confirm it.

In immunocompromised patients, especially in posttransplant patients, COVID-19 pneumonia may not be presented typically. Therefore, the high-risk population requires more careful attention [18]. In addition, clinical manifestations of COVID-19 in posttransplant patients may be uncommon due to prolonged immunosuppression [19].

Moreover, we can perform repeated oropharyngeal/nasal swabs or taking bronchoalveolar lavage if the tests are negative. However, clinical indicators like chest tomography scans can illustrate an urgent need for isolation in suspected cases [15].in immunocompromised patients; especially in posttransplant patients, COVID-19 pneumonia may not be presented typically. Therefore, the high-risk population requires more careful attention [18].

Clinical manifestations of COVID-19 in posttransplant patients may be uncommon due to prolonged immunosuppression [19]. However, in this study, CT chest [11], fever, cough, RT-PCR positive of a sample of the lavage, shortness of breath, and diarrhea are close to the symptoms in the general population of severe COVID-19 pneumonia [12, 13].

It is important to note that COVID-19 pneumonia is more severe in immunocompromised individuals than in the general nonsuppressed immune population. Guillen et al. reported kidney transplanted patients with immunocompromised that presented atypical clinical signs and symptoms such as electrolyte disorders (hyponatremia) unilobar pneumonia imaging [20].

The immunosuppressive regimen intake in this study consists of tacrolimus, mycophenolate mofetil, and prednisolone. Zhu et al. [11] describe a kidney transplant and COVID-19 that had decided several therapeutic strategies such as discontinuing all immunosuppressive agents, methylprednisolone (40 mg) daily, intravenous immunoglobulin, biapenem, and interferon ɑ (5 million units) daily. Finally, they successfully recovered their patient because of various novel treatments, but they could not figure out which medical therapy may or may not help [14]. To explain this phenomenon, Zhu et al. hypothesized that long-term suppression of the immune system might delay the clearance of the virus and prolong the course of the disease, as well as prevent the occurrence of fatal critical pneumonia due to an overimmune response [11].

On the other hand, Namazee et al. [21] reported that the discontinued immunosuppressive medications were unsuccessful in a patient with a kidney transplant with COVID-19 pneumonia. They stated that in treating a COVID-19 case with a kidney transplant, drug-drug reactions (DDI) are essential.

Therefore, the fact that we quickly used early intubation and a new treatment regimen that suppressed immune systems may help physicians develop optimal treatment strategies for similar severe cases. However, this treatment 4method requires more detailed evaluations due to the contradictory results in reviewing other studies.

## Figures and Tables

**Table 1:** Clinical laboratory outcome.

Test	Unit	Reference value	Before the illness	At the admission	At the discharge	3 weeks after discharge
*Biochemistry*						
Urea	mg/dl	Female (20-50 years): 15-40	76	181	80	61
Sodium	mEq/l	134-148	138	140	139	141
Potassium	mEq/l	3.5-5.3	3.7	4.5	4.0	3.8
LDH	U/I	Adult < 480	410	390	384	407
Albumin	g/dl	3.5-5.2	4.8	5.1	3.5	3.7
Creatinine	mg/dl	Female: 0.6-1.3	6.13	5.31	4.86	4.46
Calcium	mg/dl	Adult: 8.6-10.3	10.1	9.7	9.1	8.8
Phosphorus	mg/dl	Adult: 2.6-4.5	3.1	3.6	4.1	2.9
Magnesium	mg/dl	Female: 1.9-2.5	2.0	2.2	1.9	2.0
*Hematology*						
WBC	10^3^/*μ*l	4.0-10.1	8.4	8.7	11.5	10.0
RBC	10^6^/*μ*l	Female: 4.2-5.4	4.3	4.26	4.6	4.9
Hb	mg/dl	Female: 12-16	12.4	15.1	13.7	14.9
Hct	%	Female: 32-45	35.6	37	33.1	38.4
MCV	fl	80-96	87.6	87.3	85.6	85.3
MCH	p.g	26-32	29.5	29.1	29.6	29.8
MCHC	gr/dl	30-36	34.1	34.6	33.2	34.2
Platelets	10^3^/*μ*l	150-450	158	149	112	150
Neutrophils	%	47-76	48	47.5	31	46.8
Lymphocytes	%	Adult: 25-45	26.1	25.3	17	25.7
Monocytes	%	—	2	2	1.5	2
Eosinophils	%	—	0.05	0.5	0.00	0.06
PT patient time	Seconds	8.6-12.6	12.1	11.8	12.2	12.6
I.N.R	Seconds	0.9-1.3	1.24	1.14	1.31	1.00
PTT patient time	Seconds	20-35	29.4	23.00	29.8	21.4
*Blood gases*						
pH	—	—	7.4	7.47	7.43	7.31
P CO_2_	mmHg	—	24	25	35	33
P O_2_	mmHg	—	49	37	35	42
HCO_3_	mmol/l	—	17.5	16.6	22.7	16.6
SO_2_%	%	—	87	73	69	72
